# Toxic optic neuropathy

**DOI:** 10.4103/0301-4738.77035

**Published:** 2011

**Authors:** Pradeep Sharma, Reena Sharma

**Affiliations:** Squint and Neuro-Ophthalmology Unit, R P Centre for Ophthalmic Sciences, All India Institute of Medical Sciences, New Delhi, India

**Keywords:** Methanol, optic atrophy, scotoma, toxic optic neuropathy

## Abstract

Toxic optic neuropathy (TON) is a disease entity which is not only underdiagnosed, but also often diagnosed at a stage when recovery of vision is not possible. This article gives an overview of common causes, clinical features, and management of TON.

The anterior visual pathway is susceptible to damage from various toxins. Toxic optic neuropathy (TON) is a group of medical disorders which can be defined by visual impairment due to optic nerve damage by a toxin. It is suggested to use this term rather than “toxic amblyopia.” The exposure to a toxic substance can occur in a workplace, with ingestion of materials/foods containing a toxin, or the use of systemic medications. Both genders and all races are equally affected, and all ages are susceptible.[1]

This can also be defined as a clinical syndrome characterized by papillomacular bundle damage, central or cecocentral scotoma, and reduced color vision. Although these problems have been classified as optic neuropathies, in many of these entities, the primary lesion has not actually been localized to the optic nerve and may possibly originate in the retina, chiasm, or even the optic tracts. TON has multiple causes which are linked by shared signs and symptoms. Both toxic and nutritional factors play a synergistic role in several of these disorders.

## Signs and Symptoms

**Figure 1:**
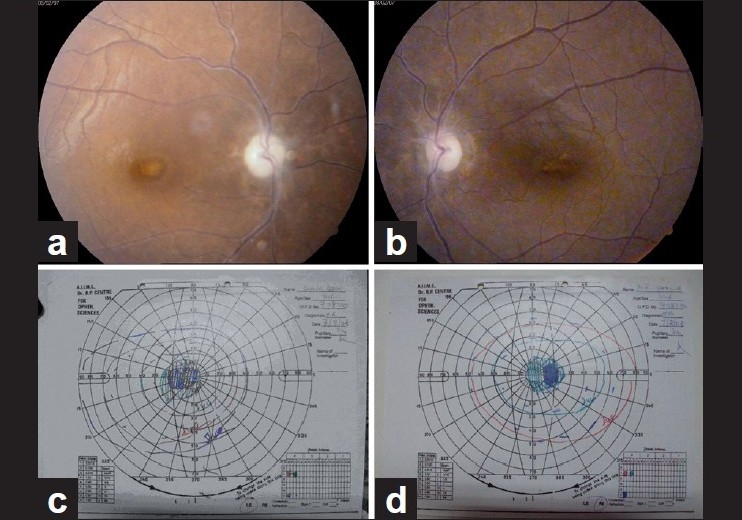
(a, b) Disc pallor in a 44-year-old female with ethambutol toxicity. She was treated with ethambutol for 2 months for tuberculoma brain. (c, d) Goldman visual fields of the same patient showing bilateral centrocecal scotomas

The condition often presents as a painless, progressive, bilateral, symmetrical visual decline with variable optic nerve head pallor[2,3] [Table 1 and Fig. 1]. Dyschromatopsia, a change in color vision, is often the first symptom. Some patients notice that certain colors, particularly red, are less bright; others have a generalized loss of color perception. This loss of color vision is out of proportion to the decline in vision.[4] Loss of visual acuity may start with a blur at the point of fixation (a relative scotoma), followed by a progressive decline (20/40-20/200).[4] The degree of vision loss can extend to total blindness, but a loss beyond 20/400 is not common, except in the case of methanol ingestion where patients can even have no perception of light. Peripheral vision is usually spared since the pattern of vision loss typically involves a central or cecocentral scotoma (usually a relative cecocentral scotoma) [Fig. 1].

**Table 1:** Clinical features of a case of toxic optic neuropathy

Symptoms
Diminution of vision: bilaterally symmetrical, painless, gradually
progressive
Dyschromatopsia
Signs
Pupils: sluggish, no RAPD
Optic disc: normal, swollen, or hyperemic in early stages:
temporal optic disc pallor later
Visual field defect: centrocaecal scotoma

**Figure 2:**
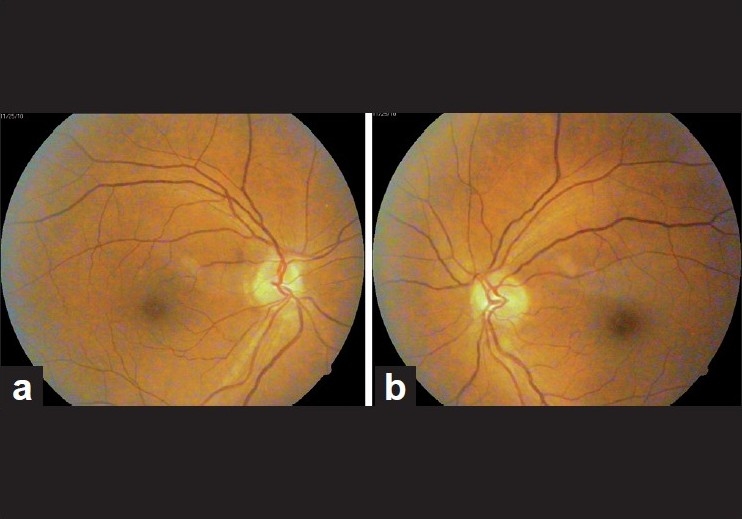
(a, b) Bilateral disc edema in a case of early chloroquine toxicity

The pupils usually demonstrate a normal response to light and near stimulation. In those who are practically blind, the pupils will be dilated with a weak or absent response to light. The optic disc may appear normal, swollen, or hyperemic in the early stages [Fig. 2].[4] Disc hemorrhages may also be present. Continued damage to the optic nerve results in the development of optic atrophy, classically seen as temporal pallor of the optic disc.

## Pathophysiology

Optic neuropathy may result from exposure to a neuro-poisonous substances toxin in the environment, ingestion of certain foods or other materials containing toxic substances, or from elevated serum drug levels. Among the many causes of TON, common ones include: ingestion of methanol (wood alcohol), treatment with disulfiram for chronic alcoholism, halogenated hydroquinolones (amebicidal medications), ethambutol and isoniazid (tuberculosis treatment), antibiotics such as linezolid and chloramphenicol and cimetidine, vincristine, and cyclosporine. Tobacco is also an important cause of TON [Table 2]. Metabolic disorders may also cause this disease. Systemic problems such as diabetes mellitus, kidney failure, and thyroid disease can cause optic neuropathy which is likely through build up of toxic substances within the body.

**Table 2:** Common causes of toxic optic neuropathy

Alcohols: Methanol, ethylene glycol (antifreeze)
Antibiotics: Chloramphenicol, sulfonamides, linezolid
Antimalarials: Chloroquine, quinine
Antitubercular drugs: Isoniazid, ethambutol, streptomycin
Antiarrhythmic agents: Digitalis, amiodarone
Anticancer agents: Vincristine, methotrexate
Heavy metals: Lead, mercury, thallium
Others: Carbon monoxide, tobacco

In most cases, the cause of the toxic neuropathy impairs the tissue’s vascular supply or metabolism. It remains largely unestablished as to why certain agents are toxic to the optic nerve while others are not and why particularly the papillomacular bundle gets affected. The unusual configuration of the vascular supply of the optic nerve head may predispose it to the accumulation of toxic agents, but this has never been proven.

Although the etiology is likely multifactorial, individuals who abuse alcohol and tobacco are at greater risk for nutritional optic neuropathy because they tend to be malnourished.[5,6] The predominant cause of nutritional optic neuropathy is thought to be a deficiency of B-complex vitamins, particularly thiamine (vitamin B1) and cyanocobalamin (vitamin B12). Deficiency of riboflavin (vitamin B2), niacin (vitamin B3), pyridoxine (vitamin B6), and folic acid also seems to play a role. Tobacco, as part of the systemic nicotine cascade, produces metabolic deficiencies.

Alcohol, like tobacco, produces its toxic effects through metabolic means. Chronic exposure typically leads to vitamin B12 or folate deficiency. Over time, these deficiencies cause accumulations of formic acid. Both formic acid and cyanide inhibit the electron transport chain and mitochondrial function, resulting in disruption of ATP production and ultimately impairing the ATP-dependent axonal transport system. Methanol causes focal retrolaminar optic nerve delamination.

The chelating properties of ethambutol have been hypothesized to contribute to its neurotoxicity. It causes a calcium flux into the mitochondria and excitotoxicity.[4,7] The mechanism of the neurotoxicity that occurs from the antiarrhythmic amiodarone remains unclear. It is believed that it may relate to a lipidosis that is induced by the drug, which has been supported by histopathologic studies of the optic nerve in these patients.[4]

All of the above risk factors impact mitochondrial oxidative phosphorylation. Thus, the toxic and nutritional optic neuropathies are actually acquired mitochondrial optic neuropathies and may behave in a similar way. The clinical picture is also similar to the congenital mitochondrial optic neuropathies.

## Workup and Investigations in a Case of TON

The diagnosis of toxic or nutritional optic neuropathy is usually established by a detailed medical history and careful eye examination. An extensive history may be the best way to uncover circumstances and situations that involve toxic neuropathy. Further testing is guided by the medical history and physical examination; and is performed to elucidate a specific toxin or nutritional deficiency as a cause of the optic neuropathy. Examples include blood testing for methanol levels or vitamin B12 levels.

Patients suspected of having a TON should have a complete hemogram, total and differential blood cell counts and urinalysis. The blood and urine also may be screened for specific toxins, especially if exposure to a particular one is not identified on history. On the other hand, if a specific intoxicant is suspected, one would try to identify it or its metabolites in the patient’s tissues or fluids. A heavy metal screening (lead, thallium) should be done based on suspicion. Serum B-12 (pernicious anemia) and red cell folate levels (marker of general nutritional status) should be obtained in any patient with bilateral central scotomas.

A complete ocular examination for evaluating TON should include color vision and visual field testing.

### Visual field examination

Visual field evaluation, static (Humphrey) or kinetic (Goldman), is absolutely essential in the evaluation of any patient suspected of having toxic/nutritional optic neuropathy. Central or cecocentral scotoma with preservation of the peripheral field are characteristic of these optic neuropathies and are actually most prevalent in patients with these disorders. Rarely, patients may present with other defects. The field defects tend to be relatively symmetric. Soft margins are another characteristic of these defects, which are easier to define/plot for colored targets, such as red, than for white stimuli.

### Neuroimaging in toxic/nutritional optic neuropathy

Although imaging studies yield normal results in toxic/nutritional optic neuropathy, they almost always are indicated, unless one is absolutely certain of the diagnosis. The most appropriate imaging study is an magnetic resonance imaging (MRI) of the optic nerves and chiasm with and without gadolinium enhancement. If the medical history is atypical or does not clearly point to a cause, neuroimaging is required to rule out other causes and confirm the diagnosis.

### Electrophysiological tests

Electrophysiological tests have also been used in patients of TON. Visual evoked response P100 wave amplitude is found to be markedly reduced with normal to near normal latency in patients of tobacco alcohol amblyopia.[8]

The detection of subclinical toxicity is rather difficult in cases of TON. Regular visual field and electrophysiological testing have been suggested for this purpose. Repeated contrast sensitivity measurement is also an easy and quicker way to detect early optic nerve damage and has especially been used in ethambutol toxicity.

### Management

The first step in managing TON, as with any toxic process, is to remove the offending agent. This may cause some reversal of the process. Treatment of TON is dictated by the cause of the disorder. Medical therapy includes vitamin supplementation which is needed in many patients with toxic neuropathy especially those with tobacco alcohol amblyopia.

Patients with toxic/nutritional optic neuropathy should be observed initially every 4–6 weeks and then, depending on their recovery, every 6–12 months. The patient’s visual acuity, pupils, optic nerves, color vision, and visual fields should be assessed at each visit. Vision gradually recovers to normal over several weeks, though it may take months for full restoration and there is always the risk of permanent residual vision deficit. Visual acuity usually recovers before color vision, the reverse of what happens at the onset of the disease process.

Morbidity of these disorders depends on the risk factors, the underlying etiology, and the duration of symptoms before the institution of treatment. A patient with advanced optic atrophy is less likely to recover visual function than a patient who does not have such pathologic changes. The prognosis is variable and depends upon the nature of the agent, total exposure prior to removal, and degree of vision loss at the time of diagnosis.

### Common causes of TON

#### Ethambutol toxicity

Ethambutol hydrochloride is a bacteriostatic antimicrobial agent used as a first-line defense against tuberculosis. The exact mechanism of action of ethambutol is unknown; however, it has been hypothesized that it acts as a chelating agent that disrupts one of the several metal-containing enzyme systems in the nucleic acid structures of mycobacteria.[8] Its toxicity involves the same mechanism. Early animal experiments showed that ethambutol causes lesions in the optic chiasm and optic nerves.[9,10] Classically described as dose- and duration-related and reversible on therapy discontinuation, reversibility of optic neuritis remains controversial. Unfortunately, patient education and immediate cessation of the drug do not always change the final visual outcome. The toxicity appears unpredictable, and therefore the drug should be used cautiously.

Ethambutol causes optic neuropathy in 1-5% of patients using the anti-tuberculous medication.[11] The dosage of 25 mg/kg/day for 2 months should be reduced to 15 mg/kg/day maintenance dose which is considered safe as well as effective, although toxicity has been reported below this dosage too.[12]

The visual symptoms usually start 2–8 months after the drug is started. Dyschromatopsia may be the earliest sign of toxicity, and blue-yellow color changes are the most common.[11] Central scotomas are the common visual field defect, but bitemporal defects and peripheral field constriction have also been reported.[3] Pupillary abnormalities can be subtle, and visual evoked potential may be needed to confirm the diagnosis.[13] Contrast sensitivity measurement has also been found effective in detecting subclinical toxicity.[14]

Optical coherence tomography (OCT), which is now commonly used to measure nerve fiber layer thickness in patients with glaucoma, can also be used to quantify such changes in ethambutol toxicity.[12] It can quantify the loss of retinal nerve fibers of these patients as a sign of early toxicity, before the fundus changes become apparent. OCT, therefore, is an additional objective test to monitor patients on ethambutol, especially when used in conjunction with visual fields.

International guidelines on prevention and early detection of ethambutol-induced ocular toxicity have been published. Nonetheless, opinion of the clinical effectiveness of regular vision tests to enable early detection of toxicity is divided. Other than stopping the drug, no specific treatment is available for the optic neuropathy caused by ethambutol. Once this is accomplished, many patients will recover, and this may take weeks to months. However, there are reports that vision may still decline or fail to recover even when the drug is stopped if damage is severe enough.[11,15]

#### Isoniazid toxicity

Isoniazid toxicity may be associated with bilateral optic disc swelling.[16] Another atypical feature is that the visual fields often take the appearance of bitemporal hemianopic scotomas. Vision improves when administration of the drug is ceased. Pyridoxine 25–100 mg/day may help stabilize or even reverse isoniazid induced toxic neuropathy. Although it has been used to reverse the toxicity of isoniazid, the improvement may simply be due to stopping the drug and not the pyridoxine.

Because both ethambutol and isoniazid may be given concurrently in the treatment of tuberculosis, and both may produce a TON, physicians should remember that if stopping one does not result in the improvement of a patient’s vision, then the other drug should also be stopped.[2]

A baseline ophthalmologic examination should be done before treatment with either ethambutol or isoniazid is instituted. This should include fundus examination, color vision, contrast sensitivity, and visual fields. They should then be monitored by their ophthalmologist periodically as long as they are on the drug to detect any optic nerve toxicity as soon as possible.

#### Methanol poisoning

Methanol toxicity remains a common problem in many parts of the developing world, especially among members of lower socioeconomic classes. It usually results from either accidental or suicidal ingestion of products containing methanol. Intoxication in industrial settings follows absorption across the skin or lung. It is metabolized by the enzyme alcohol dehydrogenase (ADH) in the liver, via formaldehyde to formic acid, the latter being responsible for the adverse effects. The toxicity develops from a combined effect of the metabolic acidosis (H+ production) and an intrinsic toxicity of the formate anion itself.

Significant ingestion causes nausea, vomiting, and abdominal pain. The central nervous system (CNS) effects of methanol resemble those of ethanol although in low doses it does not have a euphoric effect.

Formic acid accumulates within the optic nerve and causes classic visual symptoms of flashes of light. Subsequently, this may progress to scotomas and scintillations. Vision loss is thought to be caused by interruption of mitochondrial function in the optic nerve, resulting in hyperemia, edema, and optic nerve atrophy. Pupillary response to light is compromised and, subsequently, is lost.

Definitive diagnosis of methanol toxicity requires a confirmed increase in the serum methanol level with gas chromatography (>20 mg/dl). Peak levels are achieved 60–90 min after ingestion, but they do not correlate with the level of toxicity and thus are not a good indicator of prognosis. Arterial pH seems to correlate best with formate levels (<7.2 is a severe intoxication).

Supportive therapy is aimed at initiating airway management, correcting electrolyte disturbances, and providing adequate hydration. Gastric lavage is useful only if patient presents within 2 h of ingestion. Treatment consists of the usage of buffer like sodium bicarbonate to correct metabolic acidosis and an antidote to inhibit metabolism of methanol to its toxic metabolite, formic acid. If necessary, hemodialysis is supplied to further correct the acidosis, and remove both methanol and formate.

Antidote therapy is directed toward delaying methanol metabolism until it is eliminated from the system either naturally or *via* dialysis. This can be achieved by the use of either ethanol or fomepizole. Ethanol, like methanol, is metabolized by ADH, and the enzyme has 10–20 times higher affinity for ethanol compared with methanol. Fomepizole is also metabolized by the same enzyme; it has the advantage that unlike ethanol it does not cause CNS depression. However, its use is limited because of high cost and lack of availability. Ethanol is, therefore, commonly used and is given IV as a 10% solution in 5% dextrose. A loading dose of 0.6 g/kg is given followed by an intravenous (IV) infusion of 0.07–0.16g g/kg/h.

Intravenous pulse steroids have also been tried in a few patients to salvage vision, and the results have been encouraging. The benefit has been proposed to be due to anti-inflammatory and immunosuppressant effect of steroids.[17,18]

#### Tobacco–alcohol amblyopia

Tobacco–alcohol amblyopia is a condition characterised by papillomacular bundle damage, central or cecocentral scotoma, and reduction of color vision in a patient who abuses tobacco and alcohol.[19,20] This typically occurs after many years of smoking or alcohol abuse. The appearance of the optic nerve is usually normal, but peripapillary dilated vessels and hemorrhages have been described. Although this syndrome has been classified as optic neuropathy, the primary lesion has not actually been localised to the optic nerve and may possibly originate in the retina, chiasm, or even the optic tracts.[19] Vision loss may precede optic disc changes as detected by OCT in a patient with tobacco-alcohol amblyopia.[21]

Most patients with this amblyopia suffer from severe nutritional depletion, and visual improvement in these patients seems to be related to improved nutrition. It cannot, therefore, be overemphasized to patients that stopping, or at least reducing, their smoking or consumption of alcohol is critical to their recovery. The latter, combined with an improved diet (green leafy vegetables and fruit daily) and vitamin supplementation, are the mainstay of therapy. This includes institution of thiamine 100 mg orally twice daily, folate 1 mg once a day, and a multivitamin tablet daily. Injections of hydroxocobalamin have also been successful in treating patients with tobacco amblyopia, even when smoking continues.[22] Its protective effect is believed to be due to the conversion of free cyanide to cyanocobalamin by hydroxycobalamin, an analog of vitamin B12.

#### Amiodarone

Amiodarone[23] is a commonly used antiarrhythmic agent used to treat atrial and ventricular fibrillation and ventricular tachycardia. Its toxicity is associated with slowly progressive binocular loss of vision with prolonged disc swelling (over months). This is in contrast to nonarteritic ischemic optic neuropathy which can also be associated with amiodarone usage where the patient has acute unilateral disc edema resolving over several weeks. The field defect in amiodarone toxicity may be simply a generalized constriction of fields or cecocentral scotomas.

Prompt discontinuation of amiodarone (in consultation with the patient’s cardiologist) is essential if compelling evidence is there that TON is due to the drug. The visual symptoms, along with the disk swelling, can improve gradually over the next several months. Conversely, visual loss or associated field defects can persist despite having discontinued the drug, with the disc swelling progressing to optic nerve pallor. Some patients have been reported to develop disc edema and subsequent optic neuropathy even after cessation of the drug. However, it is strongly recommended to consult with the patient’s cardiologist before discontinuing the drug and it should be determined whether the less established visual complications of the drug outweigh its proven cardiac benefits.

All patients who have to be given amiodarone should have a baseline ophthalmic examination before the drug is initiated. They should subsequently be evaluated at least every 6 months. Optic nerve pathology should be excluded in patients using amiodarone even if they have the drug-induced corneal changes.

Treatment of TON is thus difficult and does not always yield fruitful results. It is, therefore, important to avoid the risk factors or detect the neuropathy at the earliest possible.

## Nutritional Optic Neuropathy

Nutritional optic neuropathy can be defined by visual impairment due to optic nerve damage secondary to nutritional deficiency. Although we are describing toxic and nutritional optic neuropathies as separate, the two have many features in common and both may coexist.

Nutritional optic neuropathy occurs mainly due to vitamin deficiency. Deficiency of thiamine (vitamin B1), cyanocobalamin (vitamin B12), pyridoxine (vitamin B6), niacin (vitamin B3), riboflavin (vitamin B2), and/or folic acid have all been implicated. The clinical presentation and basic pathophysiology are similar to TON. Most often, they present as a non-specific retrobulbar optic neuropathy.

Currently, the treatment is limited to the intensive use of vitamins with variable results in individual cases, and to the implementation of preventive measures, when feasible.

## Mitochondrial Optic Neuropathies

Leber’s hereditary optic neuropathy (LHON) and dominant optic atrophy are both nonsyndromic optic neuropathies with a mitochondrial etiology. LHON or Leber optic atrophy is a mitochondrially inherited (mother to all offsprings) degeneration of retinal ganglion cells and their axons that leads to an acute or subacute loss of central vision; this affects predominantly young adult males. This is not a TON, but may be precipitated by adverse environmental changes. At the present time, there is no effective treatment for this heredodegenerative optic neuropathy.
